# Clinical outcomes of extended versus intermittent administration of piperacillin/tazobactam for the treatment of hospital-acquired pneumonia: a randomized controlled trial

**DOI:** 10.1007/s10096-016-2819-1

**Published:** 2016-10-28

**Authors:** H. Bao, Y. Lv, D. Wang, J. Xue, Z. Yan

**Affiliations:** 1 Department of Clinical Pharmacology, Tianjin Medical University Cancer Institute and Hospital, Huan-Hu-Xi Road, Ti-Yuan-Bei, Hexi District, Tianjin, 300060 People’s Republic of China; 2 Intensive Care Unit, Tianjin Medical University Cancer Institute and Hospital, Huan-Hu-Xi Road, Ti-Yuan-Bei, Hexi District, Tianjin, 300060 People’s Republic of China; 3 Key Laboratory of Cancer Prevention and Therapy, National Clinical Research Center for Cancer, Tianjin Medical University Cancer Institute and Hospital, Huan-Hu-Xi Road, Ti-Yuan-Bei, Hexi District, Tianjin, 300060 People’s Republic of China

## Abstract

The purpose of this study was to assess the pharmacokinetic (PK) characteristics, clinical efficiency, and pharmacoeconomic parameters of piperacillin/tazobactam administered by extended infusion (EI) or intermittent infusion (II) in the treatment of hospital-acquired pneumonia (HAP) in critically ill patients with low illness severity in China. Fifty patients completed the study, with 25 patients receiving 4/0.5 g piperacillin/tazobactam over 30 min as the II group and 25 patients receiving 4/0.5 g piperacillin/tazobactam over 3 h every 6 h as the EI group. Drug assay was performed using high-performance liquid chromatography (HPLC). The percentage of the dosing interval for which the free piperacillin concentration (%*f*T) exceeds the minimum inhibitory concentration (MIC) was calculated. The patients’ therapy cost, clinical efficiency, and adverse effects were also recorded. %*f*T>MIC was about 100, 98.73, and 93.04 % in the EI arm versus 81.48, 53.29, and 42.15 % in the II arm, respectively, when the microorganism responsible for HAP had an MIC of 4, 8, and 16 mg/L. The therapy cost in the EI group was lower than that of the II group ($1351.72 ± 120.39 vs. $1782.04 ± 164.51, *p* = 0.001). However, the clinical success rate, clinical failure rate, and drug-related adverse events did not significantly differ between groups. EI treatment with piperacillin/tazobactam was a cost-effective approach to the management of HAP, being equally clinically effective to conventional II.

## Introduction

Hospital-acquired pneumonia (HAP) accounts for about 15 % of all hospital-acquired infections (HAI) and is associated with high morbidity and mortality [1]. Approximately 10 to 50 % of patients treated for nosocomial pneumonia develop antimicrobial resistance [2]. The emergence of multidrug-resistant Gram-negative organisms coupled with an alarming scarcity of new antibiotic classes has forced the healthcare community to optimize the therapeutic potential of currently available antibiotics [3].

β-Lactams, the most commonly prescribed class of antibiotics, are routinely recommended as the first-line therapy in many treatment guidelines [4]. Piperacillin/tazobactam is a β-lactam/β-lactamase inhibitor combination with in vitro activity against a broad spectrum of aerobic and anaerobic and Gram-positive and Gram-negative bacteria [5, 6]. Piperacillin/tazobactam is approved by the U.S. Food and Drug Administration (FDA) for the treatment of HAP and ventilator-associated pneumonia (VAP) [7]. For β-lactam agents, the fraction of the dosing interval during which the free drug concentrations are above the bacterial minimum inhibitory concentration (*f*T>MIC) is the pharmacodynamic (PD) index that best links drug exposure with the antibacterial effect [8]. The recommended dose for HAP is 4.5 g every 6 h (q6h), infused over 30 min, in combination with an aminoglycoside [9]. With intermittent infusion (II), β-lactams attain a high peak concentration, but a short half-life (t_1/2_) can lead to precipitous drops in plasma drug levels and a suboptimal *f*T>MIC [10]. The administration of antibiotics as a prolonged (extended or continuous) infusion has been proposed as a way to optimize pharmacokinetics (PK), resulting in more consistent plasma drug levels and maximizing *f*T>MIC, particularly for bacteria with high MIC values [11–13]. Compared with bolus dosing, increased bacterial killing is seen both in vitro and in vivo with prolonged infusions [14, 15]. In silico models suggest that exposures produced by prolonged infusions generate a greater probability of target attainment than those attained with bolus dosing [16]. However, it is unclear whether prolonged infusions of β-lactams could translate into better clinical cure or survival.

Some studies noted that clinical advantage was observed for prolonged infusion β-lactams [17–19]. Several trials to date comparing clinical cure or survival of prolonged/continuous infusion of β-lactams with II have been completed, with conflicting results [20]. A recent review summarized the evidence regarding the comparative effectiveness of prolonged versus short-term infusion of piperacillin/tazobactam, but did not synthesize the available data [21]. Most of these studies were limited to severely ill patients. We wish to add our experience with extended infusion (EI) and II of piperacillin/tazobactam in critically ill patients with low illness severity to the picture presented in the above-mentioned research. As a result, we performed a randomized, open-label, comparative clinical trial, intending to establish the clinical efficacy, PK, and pharmacoeconomic parameters of piperacillin/tazobactam administered by EI compared with II in the treatment of critically ill patients with low illness severity with HAP in China.

## Materials and methods

### Study design

This randomized, parallel study was conducted at the intensive care unit (ICU) of Tianjin Medical University Cancer Institute and Hospital, Tianjin, China. The study protocol and informed consent form were reviewed and approved by the Ethics Committee of Tianjin Medical University Cancer Institute and Hospital. This study was identified on ClinicalTrials.gov as study NCT01796717. Consent to participate was obtained from patients.

### Patients

Eligible subjects included those diagnosed of HAP based on the guidelines of the American Thoracic Society/Infectious Diseases Society of America (ATS/IDSA) [7]. All of them were aged between 18 and 70 years. Patients were excluded from entry into the study if they had severe pyemia with hypotension or/and evidences of failure of organ function (shock: systolic pressure <90 mmHg or diastolic pressure <60 mmHg, requiring more than 4 h of administration of vasopressor agents; renal impairment: urine volume <20 mL/h or <80 mL/4 h after excluding any other potentials; acute renal failure requiring dialysis; creatinine clearance (CLcr) <40 mL/min). Other exclusion criteria were: documented infection caused by pathogens beyond the antibacterial spectrum of piperacillin/tazobactam; previously diagnosed repeated lung infection (e.g., bronchial obstruction, obstructive pneumonia, pulmonary abscess, empyema, and active tuberculosis); history of allergy to penicillins; pregnancy or breast-feeding women.

### Drug administration and blood sampling

Randomization was stratified by the institution with 1:1 allocation to each arm. Following study enrollment, an unblinded research nurse or pharmacist was responsible for the preparation of the blinded medications; the allocation status was determined by opening a sequentially numbered sealed envelope. Patients were randomized to receive piperacillin/tazobactam 4/0.5 g administered either over 30 min every 6 h as the II group or 3 h every 6 h as the EI group using a syringe pump via a central venous catheter. The two dosing regimens provided equivalent total daily doses and the duration was 7–14 days. Blood samples of 4 mL were collected through intravenous access at the administration of the first dose. Time points designated for plasma drug concentration determinations were time zero (prior to the start of administration) and at 0.5, 1, 2, 3, 4, and 6 h after the start of the infusion. The samples were collected into heparinized tubes and centrifuged at 3000 rpm (4 °C) for 5 min, which were then divided into two aliquots and stored at −80 °C for later analysis.

### Drug assay

Plasma piperacillin and tazobactam concentrations were measured using high-performance liquid chromatography (HPLC), as described previously [16]. The assays were fully validated for specificity, calibration model, accuracy and precision, recovery, and stability according to the FDA guidance of bioanalytical method validation [22]. The standard curve was linear over the concentration ranges 0.1–500 mg/L (r^2^ > 0.999) for piperacillin and 0.1–200 mg/L (r^2^ > 0.999) for tazobactam, respectively. There were three quality controls for each standard curve in order to ensure assay accuracy. The within- and between-batch accuracy were within 15 % at high, medium, and low concentrations for both drugs. For piperacillin, the within-day (*n* = 5) coefficient of variation (CV) for spiked plasma control specimens of 0.2, 10, and 250 mg/L were 10.7, 8.1, and 4.6 %, respectively, and the between-day (*n* = 5) CV were 7.3, 6.5, and 3.9 %. For tazobactam, the within-day (*n* = 5) CV for spiked plasma control specimens of 0.2, 10, and 100 mg/L were 11.6, 5.8, and 6.3 %, respectively, and the between-day (*n* = 5) CV were 9.8, 3.3, and 4.2 %. Stability tests (including short-term temperature stability, long-term stability, freeze and thaw stability, and post-preparative stability) indicated that piperacillin and tazobactam remained stable and no detectable loss or degradation was observed.

The plasma concentrations versus time data were analyzed using non-compartmental methods. The DAS 2.1 PK analysis system (Anhui, China) was used to assess the PK parameters. The time to peak plasma concentration (T_max_) and peak plasma concentration (C_max_) were obtained. The elimination half-life (t_1/2_) was calculated as 0.693/Zeta (Zeta is the slope of the terminal phase). The area under the concentration–time curve (AUC) from zero to infinity (AUC_0-∞_) was equivalent to the sum of the areas from time zero to the time of the last measured concentration, calculated by using the linear trapezoidal method (until C_max_), the log-trapezoidal method (until the last measurable concentration), and the extrapolated area. The extrapolated area was determined by dividing the final measured concentration by the slope of the terminal log-linear phase. Total body clearance (CL) was calculated as dose/AUC_0-∞_. The area under the moment curve from zero to infinity (AUMC_0-∞_) was calculated using the linear trapezoidal rule and the mean residence time (MRT) was calculated as AUMC_0-∞_/AUC_0-∞_.

### Assessment of efficiency and safety

Clinical response was assessed daily by recording clinical signs and symptoms, white blood cell (WBC) count, and body temperature until it was normalized and again at the end of treatment. Clinical outcome definitions after study drug cessation, including clinical success and clinical failure, are shown in Table 1. The MIC was done according to the Clinical and Laboratory Standards Institute (CLSI) standard. The percentage of the dosing interval during which the free drug concentration exceeded the pathogen MIC (%*f*T>MIC) and four times the MIC (%*f*T>4×MIC) for individual patients was calculated for MICs of 4, 8, and 16 mg/L. The %*f*T>MIC and %*f*T>4×MIC at each MIC were calculated by using the PK parameters of piperacillin determined for each patient. We used an MIC of 16 mg/L as the clinical breakpoints for *Pseudomonas aeruginosa*, as defined by the European Committee on Antimicrobial Susceptibility Testing (EUCAST). As the drug concentration measured in this study by the HPLC method represented total (bound plus free) drug, the free drug concentration was calculated by measured the total drug concentration ×70 % [23]. Patients were monitored throughout the study for adverse events. Physical examination and laboratory evaluations were processed to confirm the presence or absence of chemical or hematological adverse events resulting from the drug.

**Table 1:** Clinical outcome definitions after study drug cessation

Clinical response	Definition
Clinical success	1. Cure or improvement of all signs and symptoms caused by the infection 2. No additional antibiotic therapy required
Clinical failure	1. Persistent or worsening of any one of the clinical symptoms 2. New clinical signs and symptoms of infection 3. Other systemic antimicrobial therapy required

### Pharmacoeconomic analysis

The costs associated with drug acquisition, preparation, administration process (including pharmacy preparation time, nursing time, cost of materials required for the drug preparation and administration, maintenance of the intravenous, site as well as waste disposal), concomitant antibiotics, and plasma drug concentration measurement using HPLC were compared in both groups.

### Statistical analysis

A sample of 50 patients was calculated to achieve a power of 85 % to detect a 20 % absolute difference in the expected outcome at a significance level of 10 %. Statistical analysis was performed using SPSS version 12.0 (SPSS Inc., Chicago, IL, USA). Quantitative variables were reported as mean and standard deviation. Comparisons between groups were performed using the Student’s *t*-test. Qualitative variables were reported as frequencies or percentages. The Mann–Whitney *U*-test was used to compare the demographic and clinical characteristics between the extended and intermittent treatment groups. Success rates were analyzed by Fisher’s exact test. The incidence of adverse events was compared by Fisher’s exact test. All statistical tests were two-tailed, and a value of *p* < 0.05 was considered to be statistically significant. The analyst was blinded to the EI or II groups.

## Results

Fifty-two patients were enrolled into the study, whereas two were withdrawn for different reasons: one patient withdrew informed consent and in the other patient, creatinine clearance diminished severely after study enrolment. Fifty patients completed the study, with 25 patients randomized to the EI group and 25 patients randomized to the II group. The baseline clinical characteristics of the study population are presented in Table 2. No statistically significant differences were found between the two groups in terms of age, body weight, body mass index (BMI), identified pathogens, CLcr, Acute Physiology and Chronic Health Evaluation II (APACHE II) score, Clinical Pulmonary Infection Score (CPIS), or procalcitonin (PCT) after bivariate analysis was performed. Similar numbers of patients in the two groups received concomitant antibiotic therapy: 3 (12 %) in the EI group and 4 (16 %) in the II group. In the continuous-infusion group, concomitant agents were aminoglycosides or vancomycin. In the intermittent-infusion group, concomitant agents were levofloxacin, ciprofloxacin, and metronidazole, in addition to aminoglycosides and vancomycin.

**Table 2:** Demographic and clinical characteristic of patients with hospital-acquired pneumonia (HAP) treated with piperacillin/tazobactam administered by extended or intermittent infusion

Characteristic	Extended infusion (*n* = 25)	Intermittent infusion (*n* = 25)
Age, years		
Mean ± S.D.	69.75 ± 5.97	67.04 ± 7.79
Median (range)	71 (56–82)	71 (56–80)
Sex, *n* (%)		
Male	14 (56)	15 (60)
Female	11 (44)	10 (40)
Body weight, kg		
Mean ± S.D.	67.12 ± 8.22	66.3 ± 8.86
Median (range)	68.5 (55.0–80.5)	65.0 (53.0–81.5)
BMI, kg/m^2^		
Mean ± S.D.	23.67 ± 3.73	22.17 ± 1.79
Median (range)	21.5 (16.3–27.9)	20.4 (17.5–30.2)
Diagnosis, *n* (%)		
Hypertension	8 (32)	12 (48)
Cardiac surgery	5 (20)	4 (16)
Cardiological	1 (4)	2 (8)
Diabetes	7 (28)	10 (40)
Tuberculosis	0 (0)	0 (0)
Infection site, *n* (%)		
Lung	10 (40)	8 (32)
Intra-abdominal	5 (20)	9 (36)
Urinary tract	6 (24)	5 (20)
Skin and soft tissue	4 (16)	3 (12)
Identified pathogens, *n* (%)		
*Pseudomonas aeruginosa*	8 (32)	5 (20)
*Escherichia coli*	7 (28)	8 (32)
*Klebsiella pneumoniae*	7 (28)	9 (36)
*Enterobacter cloacae*	2 (8)	1 (4)
*Serratia marcescens*	1 (4)	2 (8)
CLcr, mL/min (range)	73 (47–251)	79 (53–278)
Concomitant antibiotic, *n* (%)	3 (12)	4 (16)
APACHE II score		
Mean ± S.D.	23.17 ± 7.10	23.73 ± 6.73
Median (range)	18 (10–35)	20 (9–35)
CPIS		
Mean ± S.D.	7.17 ± 1.05	7.46 ± 1.30
Median (range)	7 (4–8)	7 (4–10)
PCT (μg/L)		
Mean ± S.D.	5.51 ± 1.63	3.99 ± 0.98
Median	3.31	3.56

All patients were Asian

*S.D.* standard deviation, *BMI* body mass index, *CLcr* creatinine clearance, *APACHE* Acute Physiology and Chronic Health Evaluation II, *CPIS* Clinical Pulmonary Infection Score, *PCT* procalcitonin

Plasma drug concentration sampling and PK analyses were completed on all study patients. The PK parameters of piperacillin are described in Table 3. Peak plasma concentrations of piperacillin are attained immediately after the completion of an intravenous infusion in the II group, and 2.7 ± 0.3 h after the start of infusion in the EI group. The mean peak plasma concentrations of piperacillin in the 30-min infusion group and the 3-h infusion group were 284.29 and 91.38 mg/L, respectively. Following administration, the plasma t_1/2_ of piperacillin ranged from 0.8 to 1.4 h.

**Table 3:** Main pharmacokinetic parameters of piperacillin in HAP patients after intravenous administration. Values are expressed as mean ± standard deviation (S.D.)

Parameters	Extended infusion	Intermittent infusion	*p*-Value
AUC_0-t_ (mg·h/l)	288.25 ± 47.28	433.25 ± 69.23	0.0014
AUC_0-∞_(mg·h/l)	296.91 ± 39.04	442.72 ± 43.35	<0.0001
MRT_0-t_ (h)	2.50 ± 0.31	1.55 ± 0.20	0.0002
t_1/2_ (h)	1.19 ± 0.27	1.08 ± 0.19	0.3765
T_max_ (h)	2.7 ± 0.3	0.5 ± 0.0	<0.0001
CL (L/h)	13.47 ± 1.28	9.04 ± 0.72	<0.0001
C_max_ (mg/l)	91.38 ± 20.35	284.29 ± 35.73	<0.0001

*AUC* area under the concentration–time curve, *MRT* mean residence time, *t*_*1/2*_ half-life, *T*_*max*_ time to maximum plasma concentration, *CL* clearance, *C*_*max*_ maximum observed concentration

EI regimens maintained the piperacillin concentration above a range of MICs and 4×MICs for a greater proportion of the dosing interval. A trend toward decreasing %*f*T>MIC with increasingly elevated MICs was observed. Infusion of 4 g of piperacillin, duration of 3 h over every 6 h, resulted in a mean %*f*T>MIC of about 100, 98.73, and 93.04 % for organisms with MICs of 4, 8, and 16 mg/L, respectively. Administration of 4 g of piperacillin for 30 min every 6 h resulted in a mean %*f*T>MIC of about 81.48, 53.29, and 42.15 % for MICs of 4, 8, and 16 mg/L, respectively. Significant differences in %*f*T>MIC and %*f*T>4×MIC were noted when doses were administered over an extended 3-h infusion compared with the standard 30-min infusion time evaluated in this clinical study (*p* < 0.01, Table 4).

**Table 4:** Calculated pharmacodynamic results for both groups

Parameters	Piperacillin MIC (mg/L)	Extended infusion	Intermittent infusion
%*f*T>MIC (mean ± S.D.)	4	100 ± 0	81.48 ± 3.97
	8	98.73 ± 2.31	53.29 ± 8.23
	16	93.04 ± 6.51	42.15 ± 5.56
%*f*T>4×MIC (mean ± S.D.)	4	94.29 ± 8.26	40.31 ± 4.18
	8	87.02 ± 9.70	36.35 ± 6.48
	16	73.65 ± 6.29	29.73 ± 4.21

*MIC* minimum inhibitory concentration, *%fT>MIC* and *%fT>4×MIC* percentage of the dosing interval during which free drug concentration exceeded pathogen MIC and four times the MIC

The total cost per patient in the ICU was lower in the EI group than the II group ($1351.72 ± 120.39 vs. $1782.04 ± 164.51, *p* = 0.001). There was no significant difference in the clinical failure rate between the EI and II groups (12.0 vs. 20.0 %). Furthermore, the higher probability of clinical success rate of HAP was not found by EI of piperacillin/tazobactam than by II (Table 5). There was no mortality among patients with HAP, irrespective of the MIC.

**Table 5:** Clinical outcomes of patients with HAP receiving extended or intermittent infusions of piperacillin/tazobactam

Characteristic	Extended infusion (*n* = 25)	Intermittent infusion (*n* = 25)
Clinical success rate, *n* (%)	22/25 (88.0)	20/25 80.0)
Clinical failure rate, *n* (%)	3/25 (12.0)	5/25 (20.0)
Total cost per patient in ICU, $		
Mean ± S.D.	1351.72 ± 120.39	1782.04 ± 164.51
Median	1066.17	1387.60

*ICU* intensive care unit

There was no significant difference in the safety and tolerability of both treatment groups. The incidence of study drug-related adverse events was experienced approximately by 19 (76.0 %) patients in the EI group and 23 (92.0 %) patients in the II group. Those observed drug-related adverse events with a frequency of 1 % or more in both treatment groups are presented in Table 6. The types and severity of adverse events were similar for both groups, with the most common adverse events being gastrointestinal disorders, including diarrhea, nausea, and vomiting. Other adverse effects in both treatment groups included hypokalemia, hypocalcemia, γ-glutamyltransferase increased, aspartate aminotransferase increased, alanine aminotransferase increased, thrombocythemia, eosinophil count increased, and fever, which were mild to moderate in severity. Five patients in the EI group and four patients in the II group had seriously adverse events, including renal failure, tachycardia, and confusion. Five patients withdrew from the study due to adverse events: two in the EI group (one due to a treatment-related adverse event) and three in the II group (two due to treatment-related adverse events).

**Table 6:** Drug-related adverse events in patients with HAP in either treatment group

Adverse events	Extended infusion (*n* = 25), *n* (%)	Intermittent infusion (*n* = 25), *n* (%)	*p*-Value
Gastrointestinal disorders	2 (8)	3 (12)	0.637
Hypokalemia	1 (4)	2 (8)	0.552
Hypocalcemia	1 (4)	3 (12)	0.297
γ-Glutamyltransferase increased	1 (4)	2 (8)	0.552
Aspartate aminotransferase increased	3 (12)	2 (8)	0.637
Alanine aminotransferase increased	2 (8)	1 (4)	0.552
Thrombocythemia	1 (4)	3 (12)	0.297

## Discussion

This randomized, open-label, comparative clinical trial was conducted in patients with HAP who received piperacillin/tazobactam via EI or II. Differently from studies published previously, the study presented here focused upon low illness severity patients rather than severely ill patients. To the best of our knowledge, this report is the largest study describing the PK, PD, clinical efficiency, and pharmacoeconomics of patients treated with either EI or II of piperacillin/tazobactam in China. In this study, EI or II had no impact on adequacy of treatment, although the EI group resulted in sufficient plasma concentrations. The results indicated that EI was as effective as conventional II.

A population PK study by Bulitta et al. reported that the administration of piperacillin/tazobactam by EI may be pharmacodynamically advantageous over II because the %*f*T>MIC is higher by EI [24]. The results of Monte Carlo simulations suggested that changing medical practice from bolus dosing to EI would improve target attainment rates dramatically for organisms with an MIC ≤16 mg/L [16]. Rafati et al. performed a study of 40 septic critically ill patients who received piperacillin by EI (2 g over 0.5 h as a loading dose, followed by 8 g daily over 24 h) or II (3 g every 6 h over 0.5 h). In this study, the authors found that the %T>MIC was higher by EI (100 %) than by II (62 %) when the MIC was 16 mg/L [25]. Another study conducted by De Waele et al. found that EI piperacillin/meropenem was associated with an improvement in target attainment in critically ill patients. However, a 100 % *f*T>MIC target is not reached in a significant proportion of these patients without renal dysfunction [26].

Some in vitro and animals in vivo studies on β-lactams have indicated that %*f*T>MIC is crucial for therapeutic success [9]. A recent retrospective analysis of the cephalosporin antibiotics cefepime and ceftazidime is the first data correlating PK/PD data with clinical outcome for patients. The authors found significantly improved clinical outcome when %*f*T>MIC was maintained [27]. PD modeling had also suggested a potential benefit of extended or continuous infusion of β-lactams antibiotics in clinical outcome, but this was especially noted for bacteria with high MICs, in whom conventional II may fail to achieve the PD target [25]. However, experimental studies suggested that increasing drug concentrations up to five times the MIC or more (%*f*T>5×MIC) could further optimize the bactericidal activity [15]. However, the optimum β-lactam strategy (%*f*T>MIC or %*f*T>4–5×MIC) has not yet been identified for human infections, and the threshold of efficacy is even more controversial when EI is processed [28].

In the present study of HAP patients with low illness severity, piperacillin/tazobactam administered by EI was more likely to enable better achievement of %*f*T>MIC. However, our results demonstrated that this approach did not translate into better patient outcomes. From our research, the clinical success rate and clinical failure rate were similar between groups. Drug-related adverse effects were mild to moderate and similar to studies reported in both treatment arms in other studies [29, 30]. Several observational studies with varying study designs comparing the clinical benefits of prolonged and intermittent infusion of β-lactam antibiotics have been conducted, with inconsistent results. A DALI study conducted across 68 hospitals revealed that infected critically ill patients may have adverse outcomes as a result of inadequate antibiotic exposure, and a paradigm change to more personalized antibiotic dosing may be necessary to improve outcomes for these most seriously ill patients [31]. A recent study conducted in India suggested that there was no significant difference in the clinical outcomes of patients receiving piperacillin/tazobactam via EI or II when measured by serial CPIS [32]. In a retrospective cohort study of piperacillin/tazobactam in adults with VAP caused by Gram-negative pathogens, increased clinical cure was demonstrated with MICs >8 mg/L by continuous versus intermittent infusion [29]. The results from another retrospective cohort study showed a significantly lower 14-day mortality rate by EI in patients with *Pseudomonas aeruginosa* sepsis [33]. A third retrospective cohort study, however, failed to demonstrate any improved clinical outcomes with EI of piperacillin/tazobactam [30].

The pharmacoeconomic study was specific to piperacillin/tazobactam to determine the drug supply and total labor costs associated with the acquisition, preparation, and administration of continuous and intermittent infusion regimens. Our analysis revealed that drug acquisition costs accounted for the majority of the total costs, and nursing time accounted for the largest percentage of the labor costs for both groups. In this study, EI of piperacillin/tazobactam enabled savings of about $430.32/patient to be made. Compared with II, EI was a less costly method of administration. The use of EI for the administration of β-lactams maximizes the %*f*T>MIC, allowing the use of less drug to maintain concentrations above the MIC and can decrease drug and labor consumption, leading to cost savings. In a prospective, open-label, controlled study by Grant et al., 98 hospitalized patients were randomized to receive piperacillin by continuous (12/1.5 g per day) or intermittent infusion (3/0.375 g every 6 h or 4/0.5 g every 8 h) for HAI. The authors found lower costs per patient ($399 ± 407 vs. $523 ± 526, *p* = 0.028) encompassing all costs directly related to antibiotic use (drug, preparation, treatment of adverse events, and concomitant antibiotics) [34]. A study of patients with *Pseudomonas aeruginosa* pneumonia suggested that EI of piperacillin/tazobactam enabled savings of the total daily cost of the antimicrobial by US$25–50, representing a saving of $70,000–135,000 per year on direct drug acquisition costs compared with II [33]. Results were reported in a recent study in which €15/day was saved in patients with VAP using therapeutic drug monitoring [35]. A recent research conducted by Brunetti et al. suggested that automatic substitution of EI for II piperacillin/tazobactam is safe and associated with significant cost savings. They found that the total cost per treatment course was reduced in the EI group by 13 % compared with the II group [36].

The findings of the present study demonstrated that the EI group allowed statistically lower costs compared with the II group. With the increasing interest in EI of β-lactams and the existing fiscal restraints of the healthcare system in China, further research is needed to fully analyze the pharmacodynamics, clinical efficiency, and pharmacoeconomics, in order to treat infections in both clinically sound and cost-effective fashions.

Our study has several limitations that should be taken into account when interpreting the results. First, the study design was single-blinded and it would have been enhanced by double-blinding. Second, it was a single-center study with limited sample size, limiting the applicability of the results to other ICUs; however, a pilot study was conducted to obtain preliminary data for additional studies with larger cohorts of patients. Third, this single-center study only included patients with normal renal function, which limits extrapolation of these findings to all ICU patients. Fourth, we did not investigate free drug concentrations because of the relatively low protein binding of piperacillin [23]. Instead, total drug concentrations were measured with correction for protein binding based on the literature [37]. However, changes in protein and albumin levels that occur in ICU patients may lead to unpredictable alterations in the free fraction of the drug that is active at the site of infection. Finally, some of the patients did not receive piperacillin/tazobactam as monotherapy and, consequently, the reported data may have been the results of a synergistic effect with other antimicrobials. Despite these limitations, the results of our study may contribute to an interest in the use of β-lactam antibiotics by EI. In order to achieve a more comprehensive and uniform database, a prospective, multi-center, double-blinded, comparative study is needed.

In conclusion, in this randomized, open-label, comparative study for the treatment of HAP in critically ill patients with low illness severity, the use of EI as a means of administering piperacillin/tazobactam maximized %*f*T>MIC and was more cost-effective than II of the drug. The clinical efficiency of piperacillin/tazobactam was independent of the mode of administration, either continuous dosing or intermittent dosing.
